# Adolescents’ Alcohol Use in *Botellon* and Attitudes towards Alcohol Use and Prevention Policies

**DOI:** 10.3390/ijerph17113885

**Published:** 2020-05-30

**Authors:** Elena Gervilla, Zara Quigg, Mariàngels Duch, Montse Juan, Clarisse Guimarães

**Affiliations:** 1 Balearic Islands Health Research Institute, Carretera de Valldemossa, 79, 07120 Palma, Spain; 2 Department of Psychology, University of the Balearic Islands, Carretera de Valldemossa, km 7.5, 07122 Palma, Spain; clarisseparenteguimaraes@gmail.com; 3 Public Health Institute, Liverpool John Moores University, Exchange Station, Tithebarn Street, Liverpool L2 2QP, UK; z.a.quigg@ljmu.ac.uk; 4 European Institute of Studies on Prevention, Rambla, 15 2º-3ª, 07003 Palma, Spain; mduch@irefrea.org (M.D.); mjuan@irefrea.org (M.J.)

**Keywords:** alcohol, adolescent, breath alcohol concentration, drunkenness, attitudes, intoxication, prevention policies

## Abstract

Alcohol is a common drug misused by young people worldwide. Previous studies have found that attitudes towards heavy consumption are stronger predictors than general norms concerning alcohol. This study aims to explore adolescents’ alcohol use and drunkenness, to understand adolescents’ attitudes towards alcohol use, drunkenness and prevention approaches, and to explore associations between attitudes and personal alcohol use and demographics. Methods: Cross-sectional face-to-face survey of 410 adolescents (61.2% women) who were socializing at night in the streets of Palma (Spain). Breath Alcohol Concentration (BrAC), self-reported measures of alcohol use and social variables were assessed. Results: 70.7% of respondents had a BrAC score higher than 0. The full sample reported having a mean of 3.9 drunk episodes in the last month, and a mean of 7.34 in Alcohol Use Disorders Identification Test (AUDIT). A total of 30.7% were under the minimum age limit for alcohol drinking in Spain and males showed higher BrAC than females. Bivariate analyses identified some differences in attitudes across participant demographics and personal alcohol use. In conclusion, we found high levels of alcohol use and drunkenness amongst adolescents, and adolescents’ attitudes towards drunkenness and prevention approaches were associated with their alcohol consumption as well as with age.

## 1. Introduction

Globally, alcohol is one of the most common drugs misused by young people [1]. Whilst young people may drink less frequently than adults, they tend to do so in larger quantities over a shorter period of time, often referred to as binge drinking or heavy episodic drinking [2]. This type of heavy drinking has been shown to peak in late teenage years and early adulthood [3] and is of particular public health concern.

A broad range of alcohol-related individual and social problems are often linked to alcohol intoxication. From the individual perspective, harmful use of alcohol is one of the key risk factors for the development of mental health conditions [4] like Alcohol Use Disorders (AUD), one of the most prevalent mental disorders. The diagnosis of AUD includes alcohol dependence and alcohol abuse in the *Diagnostic and Statistical Manual of Mental Disorders* (DSM-5) [5] or harmful use in the *International Classification of Diseases and related health problems* (ICD-10) [6]. The risk of development of AUD increases with the frequency of binge drinking and is linked to higher mortality risk in younger age groups [7]. From a social perspective, as well as damaging the health of drinkers, particularly in young people whose bodies are still developing, such drinking behaviours might involve harms on others through violence or noise and disorder in public spaces, and place major burdens on health and criminal justice systems [8,9].

The European Region has the highest levels of alcohol consumption in the world and subsequently high proportions of alcohol attributable ill health and premature death [10]. Levels of alcohol consumption vary widely across European countries however, and studies suggest an increase in the proportion of young people abstaining from alcohol consumption [11]. In Spain, data from 2016 suggest that the proportion of young people (aged 15–24 years) abstaining from alcohol consumption was higher than in 2010, and higher than that of young people in other EU countries [11]. Similarly, prevalence of heavy episodic drinking was lower, both compared to 2010 and other EU countries [11]. However, studies suggest that harmful alcohol use amongst young people is a key concern across Spain [12,13]. For example, a study on nightlife users in Palma found that 60% of participants preloaded prior to entering pubs/bars or nightclubs (including drinking at home or in public spaces, known as *botellon*), and females drank 7 standard alcoholic drinks and males 9 drinks during the course of a night out [14]. Moreover, a study carried out in nine European cities showed that recreational drug use and binge drinking increased the chances for unsafe and regretted sex [15]. With evidence illustrating strong links between heavy alcohol use and harms such as violence and injury, there is a need to prevent the development of cultures of intoxication amongst Spanish youth, to limit risks to the population’s health. However, to do this effective policy makers and practitioners require information on the issue to develop effective prevention and response strategies.

Studies on young people’s alcohol consumption suggests that alcohol is a central part of socialising for young people, increasing relaxation, confidence and social interaction [16]. Getting drunk is expected to be fun, associated with pleasure and laughter [17]. In some cultures (e.g., United Kingdom), extreme drunkenness is a desired goal [18], yet elsewhere drunkenness is less acceptable, with drinkers maintaining a threshold of happy drunkenness throughout a night out as opposed to an increase in intoxication levels [14]. In young people in particularly, studies illustrate relationships between attitudes, expectations, social norms and alcohol use [18,19].

Attitudes can provide relatively stable evaluative judgments of various aspects of a person’s experience [20] and represent a key explanatory variable in many theories of health behaviour [21] because they predict both intentions and one’s behaviour [22,23]. Attitudes are constructs referring to individual dispositions to respond favourably or unfavourably to any aspect of the individual’s world, including cognitive, affective and behavioural components [24]. In this sense, Alcohol Use Disorders Identification Test (AUDIT) [25] uses 10 questions related to the three components: cognitive (e.g., ‘How often during the last year have you been unable to remember what happened the night before because you had been drinking?’), affective (e.g., ‘How often during the last year have you had a feeling of guilt or remorse after drinking?’) and behavioural (e.g., ‘How often do you have six or more drinks on one occasion?’) to assess alcohol use.

Alcohol expectations, on the other hand, can be seen as components of attitudes towards alcohol and refer to beliefs about the cognitive, affective or behavioural effects of alcohol use that can be positive or negative. Expecting positive effects is associated with increased alcohol use and alcohol problems among adolescents [26] and college students [27]. However, positive alcohol expectancies could also have an indirect effect on alcohol use and alcohol problems [28] and interact with other factors (e.g., self-efficacy for avoiding heavy drinking in social situations) [29]. In addition, longitudinal research has demonstrated that adolescents with more positive alcohol expectancies reported greater alcohol use and a higher likelihood of alcohol misuse in adulthood [30].

Finally, adolescent and young people’s drinking is heavily influenced by perceived social norms and acceptance of alcohol use is highly correlated with underage drinking [31]. Previous studies have found that attitude towards heavy consumption is a stronger predictor than norms [23]. Other authors have found that favourable attitudes towards alcohol use and getting drunk have strong associations with drinking intention and frequency [23,32,33]. In this sense, gender differences have been found, indicating that compared to women, men have more pro-alcohol attitudes and they seem to be related to greater alcohol consumption [23]; while older people and moderate drinkers were more supportive of alcohol control policies than heavy drinkers and younger people, who were more reluctant to accept intrusive alcohol policies [34,35]. Public attitudes towards alcohol policies can provide knowledge on possible implementation and adherence to interventions that count with public support [34,36,37]. Most studies assessing attitudes towards alcohol use and alcohol policies have used adult people or young adults as samples. However, attitudes perform differently in adolescents and young adults [38].

The objectives of this study are (a) to explore adolescents’ alcohol use and drunkenness, (b) to understand adolescents’ attitudes towards alcohol use, drunkenness and prevention approaches and (c) to explore associations between attitudes and personal alcohol use and demographics.

## 2. Materials and Methods

We ran a cross-sectional face-to-face survey of 410 adolescents who were participating in *botellon* (open-air gatherings of binge-drinkers) in the Balearic Islands (Spain).

The current study is part of a larger project [39]. We selected a convenience sample of adolescents (range 14 to 19 years old) who were participating in *botellon* in the city of Palma while they were socializing. Once they were selected, researchers approached them and asked for verbal informed consent to participate. *Limesurvey* mobile app [40] was used to administer the questionnaires and collect data. Participants were anonymously interviewed in order to guarantee respondents’ privacy.

Participants answered a brief interview on demographics (gender, age), attitudes towards drunkenness and prevention approaches (being 1 = strongly agree and 5 = strongly disagree) and past month frequency of drunkenness. Breath Alcohol Concentration (BrAC) (mg/L) was assessed with a breathalyzer (Zaphir CDP 3500BT). Adolescents were also asked to complete the questions of the Alcohol Use Disorders Identification Test (AUDIT) [25]. Finally, we asked the amount of time that adolescents had spent in the public setting until the moment of the interview.

Teams of two to five researchers went to the public spaces on Thursday, Friday and Saturday nights. Participants were asked to rinse their mouth with water before assessing the objective measure (BrAC) in order to eliminate any residual alcohol traces. There were no financial incentives for adolescents. Ethical approval was obtained from the ethics committee of University of Balearic Islands (approval number 75CER18).

Before approaching participants, researchers assessed their level of drunkenness based on how steady they were on their feet, whether they were swaying and how loud they were talking [41]. Interviewers were instructed not to approach extremely intoxicated or aggressive individuals (or those who seemed unable to answer questions due to alcohol use) and to call the emergency services when an adolescent showed signs of alcohol poisoning.

## 3. Results

Participants had a mean age of 17.8 years (SD = 1.3) and 61.2% were women. The median length of time in the drinking setting at the time of the interview was 52 minutes (IQR = 65). 70.7% of respondents had a BrAC score higher than 0. The mean BrAC level at time of the interview was 0.18 mg/L (SD = 0.24). The full sample reported having a mean of 3.8 drunk episodes in the last month, and a mean AUDIT score of 7.3 (44.2% AUDIT risk).

**Figure 1:**
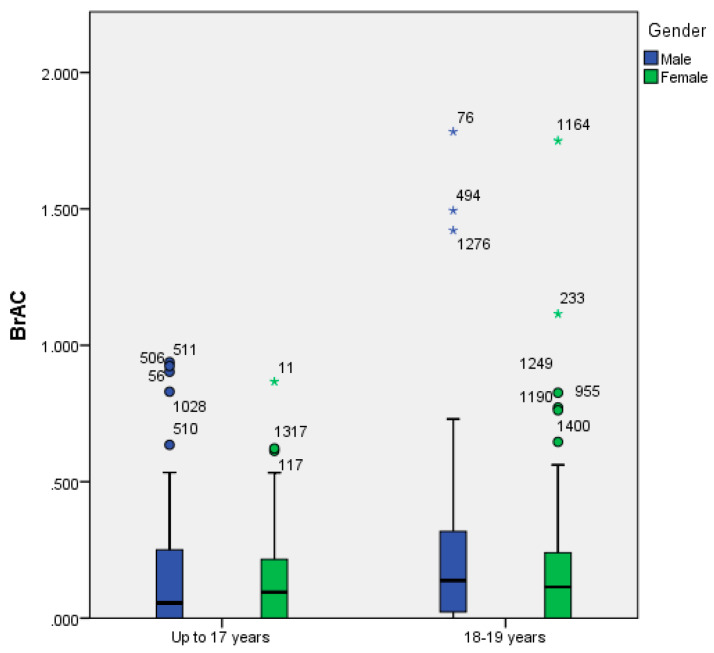
Boxplot of Breath Alcohol Concentration (BrAC) level by age group for alcohol drinking in Spain (up to 17 years of age and 18 or more years of age) and gender (male and female).

There was no significant difference by gender in proportion of adolescents with BrAC > 0 (χ^2^ = 0.014; df = 1; *p* = 0.905), risky AUDIT (χ^2^ = 0.490; df = 1; *p* = 0.484), minutes in the public setting (t = 0.436; df = 373; *p* = 0.663), number of alcoholic drinks (t = 0.059; df = 382; *p* = 0.953), drunk episodes in the last month (t = 0.734; df = 377; *p* = 0.463) or AUDIT score (t = 0.219; df = 240; *p* = 0.827). However, there was a significant difference in BrAC level by gender (t = 2.006; df = 261.960; *p* = 0.046) meaning that BrAC level for males was higher (0.22 mg/L) than for females (0.16 mg/L) (see Figure 1).

Moreover, 30.7% of the sample were under the minimum age limit for alcohol drinking in Spain which is 18 years. Therefore, differences in the sample by the legal age to drink alcohol in Spain were also explored. No significant differences were found between underage participants (up to 17 years) and adolescents with the legal age to drink alcohol (18–19 years) regarding gender, proportion of participants with BrAC > 0, risky AUDIT score, mean BrAC level at the moment of the interview, average number of alcoholic beverages, mean drunk episodes in the last month or average AUDIT scores (see Table 1). However, we found a significant difference in the average time spent in the drinking setting at the time of the interview: older adolescents had spent more time in the public setting at the time of the interview.

The majority of participants agreed that “not providing more alcohol to people who are already drunk would improve nights out” of their companions (70.8%) and that “drunk people should not be able to obtain more alcohol” (58.6%); 49.2% disagreed that “a good night out means getting drunk”. Around four in ten agreed that “drunk people ruin a night out” of their companions (43.5%), although there were differences by gender. Equal proportions agreed (36.7%) or disagreed (37.1%) that “drunk people should be able to enter nightlife venues”. There were mixed views on whether “it is acceptable for people under 18 years of age to buy or be bought alcohol”.

Bivariate analyses identified some differences in attitudes across participant demographics and personal alcohol use. Responses to the statement “drunk people ruin a night out” differed significantly by gender, AUDIT score and BrAC level; whilst agreement with the statements “drunk people should not be able to obtain more alcohol” varied by BrAC level, drunk episodes in the last month and AUDIT score. Agreement with the statements “a good night out means getting drunk” varied by age, BrAC level, drunk episodes in the last month and AUDIT score. Additionally, “drunk people should be able to enter nightlife venues” varied by gender, drunk episodes in the last month and AUDIT score. Finally, agreement with the statement “It is acceptable for people under 18 years of age to buy or be bought alcohol” varied by age and AUDIT score. Results are shown in Table 2.

In adjusted analyses (see Table 3), a selection of attitudes was significantly associated with alcohol use and drunkenness, and age, but not gender. Thus, those with a high-risk AUDIT score had significantly higher odds of agreeing with the statements “a good night out means getting drunk” (adjusted odds ratio (AOR) 2.7, *p* = 0.011). As age increased, agreement with the statements “a good night out means getting drunk” (AOR 0.6, *p* = 0.001), “it is acceptable for people under 18 years of age to buy or be bought” (AOR 0.7, *p* < 0.01) and “drunk people ruin a night out” (AOR 0.8, *p* = 0.039) decreased. As BrAC level increased, agreement with the statement “drunk people should not be able to obtain more alcohol” (AOR 0.2, *p* = 0.010) decreased. The odds of agreeing with the statement “drunk people should be able to enter nightlife venues” increased as the number of drunk episodes increased (AOR 1.1, *p* = 0.006) while the odds of agreeing with the statement “drunk people should not be able to obtain more alcohol” decreased as the number of drunk episodes increased (AOR 0.9, *p* = 0.003).

## 4. Discussion

The aim of the current study was to explore adolescents’ alcohol use and drunkenness, to understand adolescents’ attitudes towards alcohol use, drunkenness and prevention approaches and to explore associations between attitudes and personal alcohol use and demographics.

Firstly, we found high levels of alcohol use and drunkenness amongst the adolescents’ sample. The average BrAC at time of the interview was 0.18 mg/L and 70% of adolescent respondents had been drinking alcohol according to the BrAC assessments. Furthermore, self-reported measures showed that 44.2% indicated an AUDIT score of risk and an average of almost 4 drunk episodes in the last month. Average AUDIT score was 7.3, a value which almost doubled the threshold to consider the AUDIT score to be risky in adolescents.

Interestingly, while we did not find a significant difference by gender in self-reported measures of alcohol use, objective measurements of alcohol use (BrAC level) for males was higher than for females. Importantly, 30.7% of the sample were under the minimum age limit for alcohol drinking in Spain which is 18 years. Younger adolescents and adolescents with the legal age to drink in Spain (18 years) did not differ in gender, objective and self-reported alcohol use. To dispose of objective and self-reported data of alcohol use, specially binge drinking, is important in order to prevent potential consequences on mental health (e.g., Alcohol Use Disorders), mortality risk and/or unsafe and regretted sex [15], especially in younger participants.

Secondly, adolescents generally agreed that limiting access to alcohol to drunk people would improve nights out and half of them disagreed that a good night out is related to getting drunk. More than 40% agreed that drunk people ruin a night out. There were mixed views on whether it is acceptable for adolescents under the legal age to buy alcohol or whether drunk people should enter nightlife venues. Further analysis indicated that attitudes were related to gender and objective and self-reported measures of alcohol use.

Thirdly, odds ratio indicated that adolescents’ attitudes towards drunkenness and prevention approaches were associated with their alcohol consumption as well as with age but not with gender. In this sense, those with a high-risk self-reported alcohol use had significantly higher odds of agreeing with the statements “a good night out means getting drunk” while those with a lower level of alcohol use were more keen to accept prevention policies regarding alcohol use. As age increased, agreement with the statements “a good night out means getting drunk”, “it is acceptable for people under 18 years of age to buy or be bought” and “drunk people ruin a night out” decreased. In line with previous studies, these results indicate that people who do not drink tend to be more supportive of alcohol policies while those who use alcohol, and thus are more affected by the regulations, are less supportive [42,43,44].

In line with other studies, we found that favourable attitudes towards alcohol use and getting drunk have strong associations with alcohol use [23,32,33]. These results suggest that adolescents’ attitudes towards alcohol use could be addressed in programs aiming to prevent and/or reduce risky levels of drinking among youngsters [37,45,46,47,48]. Furthermore, this innovative data provides relevant information for the establishment of municipal policies from an environmental and community approach. Heavy alcohol drinking in open-air public spaces has serious health and safety implications for participants and surrounding communities that need to be addressed. Legislation on alcohol and the use of public environments should be reviewed to identify gaps and ameliorate enforcement methods. In addition, sensitization strategies should be implemented to raise community awareness and community mobilization towards adolescents and youngsters’ alcohol use prevention. In this sense, strengthening the public’s beliefs on policy effectiveness and promoting positive attitudes towards alcohol-related policies would increase public support for more restrictive alcohol control approaches and might help to reinforce their implementation.

The current study advances knowledge in the relationship between adolescents’ attitudes towards alcohol use and objective and subjective assessment of alcohol use in a natural setting. Moreover, a big sample of adolescents were interviewed, which allowed us to analyse the attitudes of adolescents who, besides, did not always have the legal age to use alcohol in Spain. Other authors have used objective measures of alcohol consumption [49] or have assessed opinions on alcohol use and on prevention policies for reducing alcohol intoxication in other public settings, like football matches [42]. However, to our knowledge, this is the first study to assess alcohol intoxication through both objective and self-reported measures of alcohol use among adolescents who are socializing in public settings and assessing their attitudes toward alcohol policies at the same time.

Our study has limitations. The survey questionnaire was short due to the natural setting and some potentially relevant factors that could be related to alcohol use, could have not been assessed. In addition, factors like tolerance, that can influence alcohol absorption, were not assessed. Moreover, this is a cross-sectional study, and the results have to be interpreted in terms of relationships but not causality. Another limitation was that research staff avoid interviewing participants who were very drunk or could be violent. This could potentially have produced lower BrAC levels than would have been obtained if all individuals found in the public setting had been interviewed. Finally, it is important to highlight the potential impact of alcohol use on the cognitive possibilities of participants and, thus, the responses given to the self-reported test AUDIT. However, the strengths include the assessment of alcohol use with objective and self-reported measures and the training of research staff prior to data collection.

## 5. Conclusions

We found high levels of alcohol use and drunkenness amongst adolescents. In addition, adolescents’ attitudes towards drunkenness and prevention approaches are associated with their alcohol consumption as well as with age but not with gender. Older adolescents and those with a lower level of alcohol use were more keen to accept prevention policies regarding alcohol use.

This study adds knowledge about the level of alcohol intoxication and attitudes among youngsters practicing binge-drinking in open-air public spaces and can inform researchers, public health officials, policy and decision makers and the general public.

## Figures and Tables

**Table 1:** Differences by age in demographics and alcohol use.

Caption	Full Sample (*n* = 410)	<18 Years (*n* = 126)	≥18 Years (*n* = 284)	*p*
Gender (Female)	61.2%	62.7%	60.6%	0.682
BrAC > 0	70.7%	65.9%	72.9%	0.150
Risky AUDIT score	44.2%	46.4%	43.4%	0.669
Expected a lower BrAC level	20.3%	14.0%	22.9%	0.222
BrAC level at interview (mean, SD)	0.18 (0.24)	0.16 (0.21)	0.20 (0.25)	0.174
Minutes spent in the public setting at the time of interview (mean, SD)	72.5 (76.8)	58.0 (52.7)	78.5 (84.2)	0.005
Number of alcoholic beverages (mean, SD)	2.7 (3.9)	3.1 (5.3)	2.5 (3.0)	0.270
Drunk episodes in the last month (mean, SD)	3.8 (5.0)	3.3 (5.7)	4.1 (4.7)	0.203
AUDIT scores (mean, SD)	7.3 (4.4)	7.6 (5.1)	7.3 (4.1)	0.240

Note: BrAC: Breath Alcohol Concentration; AUDIT: Alcohol Use Disorders Identification Test.

**Table 2:** Bivariate analysis to compare attitudes by demographics and personal alcohol use.

Caption			Gender			Age		BrAC		Drunk Episodes in The Last Month		Audit Score	
		All %	Female %	Male %	*p*	Mean (SD)	*p*	Mean (SD)	*p*	Mean (SD)	*p*	Mean (SD)	*p*
Full sample		NA	61.2	38.8	NA	17.8 (1.3)	NA	0.2 (0.2)	NA	3.8 (5.0)	NA	7.3 (4.4)	NA
A good night out means getting drunk	Agree	21.9	21.9	22.1	0.441	17.3 (1.5)	0.007	0.3 (0.3)	0.027	5.2 (4.9)	0.003	9.8 (4.9)	<0.001
	Neutral	28.9	31.4	24.8		17.9 (1.2)		0.2 (0.2)		4.4 (6.3)		7.9 (4.1)	
	Disagree	49.2	46.8	53.1		18.0 (1.3)		0.2 (0.2)		3.0 (3.8)		5.9 (3.7)	
It is acceptable for people under 18 years of age to buy or be bought alcohol	Agree	25.4	25.0	26.1	0.605	17.3 (1.3)	<0.001	0.2 (0.2)	0.287	4.7 (4.7)	0.216	9.0 (4.8)	0.006
	Neutral	27.8	26.1	30.6		18.0 (1.2)		0.2 (0.2)		4.0 (6.3)		7.3 (4.8)	
	Disagree	46.7	48.9	43.2		18.0 (1.4)		0.2 (0.3)		3.4 (4.1)		7.4 (4.4)	
Drunk people ruin a night out	Agree	43.5	45.1	41.1	0.006	17.8 (1.4)	0.469	0.2 (0.3)	0.025	3.5 (5.4)	0.200	6.5 (4.0)	0.017
	Neutral	30.6	35.2	23.2		17.9 (1.2)		0.2 (0.2)		4.1 (4.9)		7.7 (4.2)	
	Disagree	25.9	19.8	35.7		18.0 (1.2)		0.2 (0.3)		4.8 (4.6)		8.4 (4.7)	
Drunk people should be able to enter nightlife venues	Agree	36.7	35.9	38.1	0.047	18.0 (1.2)	0.061	0.2 (0.3)	0.114	5.6 (6.7)	<0.001	8.5 (5.0)	0.007
	Neutral	26.2	30.9	18.6		18.0 (1.4)		0.2 (0.3)		3.6 (3.7)		6.6 (3.5)	
	Disagree	37.1	33.1	43.4		17.6 (1.3)		0.2 (0.2)		2.7 (3.2)		6.7 (3.8)	
Drunk people should not be able to obtain more alcohol	Agree	58.6	62.1	53.1	0.108	17.8 (1.3)	0.827	0.2 (0.2)	0.003	3.0 (3.6)	0.002	6.6 (4.1)	0.002
	Neutral	18.6	19.2	17.7		17.8 1.5)		0.2 (0.2)		5.2 (5.9)		8.1 (4.5)	
	Disagree	22.7	18.7	29.2		17.8 (1.3)		0.3 (0.3)		5.5 (6.7)		9.0 (4.9)	
Not providing people who are already drunk with more alcohol would improve nights out	Agree	70.8	72.2	68.4	0.667	17.8 (1.4)	0.391	0.2 (0.3)	0.111	3.7 (4.6)	0.103	7.2 (4.5)	0.457
	Neutral	17.3	17.1	17.5		18.0 (1.2)		0.2 (0.2)		5.3 (7.0)		7.6 (3.8)	
	Disagree	12.0	10.7	14.0		17.8 (1.3)		0.3 (0.2)		3.5 (3.1)		8.3 (4.8)	

**Table 3:** Adjusted analyses (odds ratios) of the relationship between attitudes, demographics and alcohol use, controlling by the amount of time spent in the public setting.

Caption		Risk AUDIT	Female	Age	BrAC	Drunk Episodes
A good night out means getting drunk	AOR	2.7	1.0	0.6	3.9	1.0
	*p*	0.011	0.932	0.001	0.060	0.270
It is acceptable for people under 18 years of age to buy or be bought alcohol	AOR	1.7	1.1	0.7	2.8	1.1
	*p*	0.175	0.845	<0.001	0.153	0.144
Drunk people ruin a night out	AOR	0.8	1.1	0.8	0.8	0.9
	*p*	0.528	0.701	0.039	0.790	0.053
Drunk people should be able to enter nightlife venues	AOR	1.4	0.9	1.3	1.8	1.1
	*p*	0.283	0.679	0.054	0.349	0.006
Drunk people should not be able to obtain more alcohol	AOR	0.9	1.2	1.2	0.2	0.9
	*p*	0.732	0.560	0.180	0.010	0.003
Not providing people who are already drunk with more alcohol would improve nights out	AOR	0.8	1.3	0.9	0.5	1.0
	*p*	0.575	0.417	0.487	0.305	0.487

## Abbreviations

BrAC	Breath Alcohol Concentration
AUDIT	Alcohol Use Disorders Identification Test
AUD	Alcohol Use Disorders
DSM-5	Diagnostic and Statistical Manual of Mental Disorders
ICD-10	International Classification of Diseases and related health problems
AOR	adjusted odds ratio
